# Ruptured Tubal Ectopic Pregnancy at Fifteen Weeks Gestational Age

**DOI:** 10.5811/cpcem.2019.1.40860

**Published:** 2019-01-29

**Authors:** Justine K. Stremick, Kyle Couperus, Simeon W. Ashworth

**Affiliations:** * Fort Belvoir Community Hospital, Department of Emergency Medicine, Fort Belvoir, Virginia; † Madigan Army Medical Center, Department of Emergency Medicine, Joint Base Lewis-McChord, Washington

## Abstract

Tubal ectopic pregnancies are commonly diagnosed during the first trimester. Here we present a second-trimester tubal ectopic pregnancy that was previously misdiagnosed as an intrauterine pregnancy on a first-trimester ultrasound. A 39-year-old gravida 1 para 0 woman at 15 weeks gestation presented with 10 days of progressive, severe abdominal pain, along with vaginal bleeding and intermittent vomiting for two months. She was ultimately found to have a ruptured left tubal ectopic pregnancy. Second-trimester ectopic pregnancies carry a significant maternal mortality risk. Even with the use of ultrasound, they are difficult to diagnose and present unique diagnostic challenges.

## INTRODUCTION

While ectopic pregnancies account for only 1.3–2.4% of all pregnancies, they are the leading cause of first-trimester maternal pregnancy-related mortality and account for 10% of maternal pregnancy-related deaths.^1^,^2^ Ninety-five percent of ectopic pregnancies occur in the fallopian tubes, and these are most often discovered in the first trimester. There are only a handful of cases of second-trimester tubal ectopic pregnancies published in the literature.^3^ Most ectopic pregnancies are diagnosed between six and nine weeks of gestation when the patient becomes symptomatic.^1^ They often present with complaints of vaginal bleeding, pelvic pain and occasionally syncope.^3^ Tubal rupture is common with as many as 16% of tubal ectopic pregnancies showing signs of rupture by six weeks of gestational age.^1^ Here we present a rare case of a tubal ectopic pregnancy that progressed to 15 weeks after being misdiagnosed as an intrauterine pregnancy during a first-trimester ultrasound.

## CASE REPORT

A 39-year-old gravida one para zero woman at an estimated 15 weeks four days gestation presented to the emergency department (ED) with 10 days of progressive, severe abdominal pain. She also reported moderate vaginal bleeding for the prior several months along with intermittent nausea and vomiting. Six weeks prior to presentation, she was seen in clinic where her obstetrician performed a point-of-care ultrasound (POCUS). It was documented that the patient had an intrauterine pregnancy with an estimated gestational age of nine weeks and zero days.

**Image 1:**
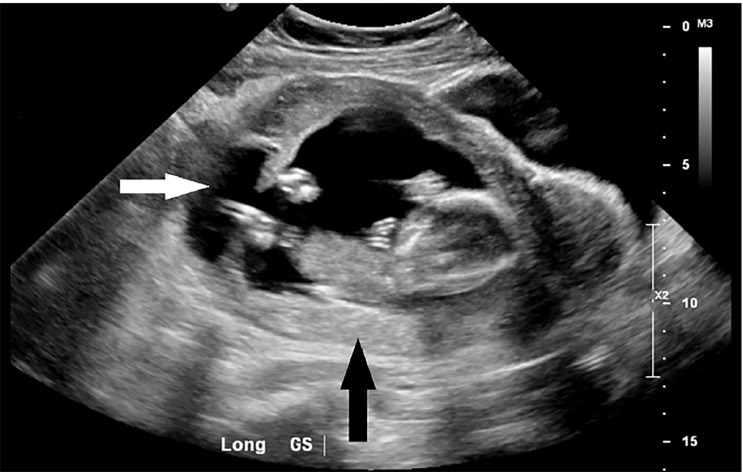
Longitudinal image of the gestational sac (GS) depicted by the black arrow. A communication between the gestational sac and peritoneum is depicted by the white arrow.

In the ED she was tachycardic to 131 beats per minute and normotensive at 116/84 millimeters of mercury. Her exam was significant for tenderness to palpation of her entire abdomen with rebound and guarding present. Her labs were significant for a moderate anemia with a hemoglobin of 9.2 grams per deciliter along with a leukocytosis of 13,200 white blood count per millimeter cubed, and mild elevations of her alanine aminotransferase and aspartate aminotransferase at 76 units per liter (u/L) and 53 u/L, respectively. Limited POCUS identified a fetus measuring 16 weeks one day by biparietal diameter with a heart rate of 163 bpm. Oligohydramnios was noted. Obstetrics was consulted at that time. Shortly afterwards, the patient was taken for a formal ultrasound. This showed free fluid and clotted blood throughout her abdomen and was initially concerning for uterine rupture (Image 1).

The patient was taken to the operating room for an exploratory laparotomy and was found to have a ruptured left tubal ectopic pregnancy. She underwent a left-sided salpingo-oophorectomy and required four units of packed red blood cells. She did well post-operatively and was discharged home on post-operative day two.

## DISCUSSION

Vaginal bleeding, pelvic and abdominal pain occurring in the first 20 weeks of pregnancy are common ED complaints. The differential is broad, including both obstetric and non-obstetric causes, and diagnosis can often be complicated by displacement of intra-abdominal organs. During the work-up of these patients, one must maintain a high clinical suspicion for ruptured ectopic pregnancy, even in the setting of prior imaging showing an intrauterine pregnancy. This can either be due to a heterotopic pregnancy or, as in this case, an initial misdiagnosis of an ectopic pregnancy as an intrauterine pregnancy.

Ultrasound plays a critical role in the work-up and diagnosis of ectopic pregnancy. Typical ultrasound features used to diagnose ectopic pregnancies in the first trimester include the presence of a pseudo-gestational sac, thickened endometrium, fluid in the posterior cul-de-sac and the tubal ring sign. The tubal ring sign is the most specific finding for a tubal ectopic pregnancy but may become less reliable as the pregnancy advances and the tubal wall thins.^3^

The most common type of ectopic pregnancy that is mistaken for an intrauterine pregnancy during first trimester ultrasound is an interstitial pregnancy. Interstitial ectopic pregnancies are located in the fallopian tube just as it meets the uterine cavity, with most of the gestational sac located outside of the uterus. Ultrasound findings that are suggestive of interstitial pregnancy include a gestational sac covered by an asymmetric or incomplete myometrial mantle, an empty uterine cavity with a central linear echo, an eccentrically located gestational sac, myometrium located between the gestational sac and uterine cavity, and a gestational sac seen high in the uterine fundus.^4^

**CPC-EM Capsule**

What do we already know about this clinical entity?

*Ectopic pregnancies are a leading cause of maternal morbidity and mortality that rarely progress to the second trimester*.

What makes this presentation of disease reportable?

*This is a rare case of a tubal ectopic pregnancy that progressed to 15 weeks gestational age after being initially misdiagnosed as an intrauterine pregnancy during a first-trimester ultrasound*.

What is the major learning point?

*Although ultrasound has revolutionized the diagnosis of ectopic pregnancy, its use can sometimes mislead practitioners and in general present unique diagnostic challenges*.

How might this improve emergency medicine practice?

*This article reviews common ultrasound findings that can help distinguish later-term ectopic pregnancies from normal intra-uterine pregnancies, aiding in quicker recognition and diagnosis*.

Unfortunately, images from this patient’s initial in-office ultrasound were not saved and therefore not available for review. However, the surgical report does not indicate that this was an interstitial pregnancy. For other types of tubal ectopic pregnancies, transvaginal sonography has a 99.9% specificity in diagnosing ectopic pregnancies in the first trimester; so it is likely that human error played a role in her misdiagnosis.^3^

**Image 2:**
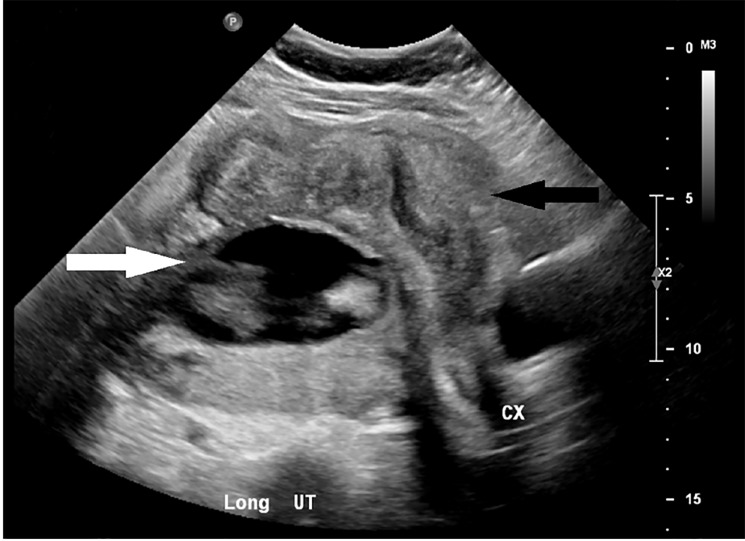
Longitudinal image of the uterus (UT) is depicted by the black arrow. The gestational sac is seen sitting posterior to the uterus and is depicted by the white arrow. *CX*, cervix.

Later-term ectopic pregnancies can present unique challenges and are often misdiagnosed. Common ultrasound findings suggestive of an ectopic pregnancy are an abnormal fetal lie, displaced cervix, oligohydramnios, and maternal intraperitoneal fluid.^5^ On further review of the formal ultrasound imaging in this case, the gestational sac can be seen lying posterior to the uterus (Image 2). Echogenic fluid may not be seen in the posterior cul-de-sac in up to two-thirds of tubal pregnancies, as was true in this case. It is therefore prudent to check for either non-echogenic fluid, representing clotted blood in the posterior cul-de-sac, or fluid in the right upper quadrant.^1^

## CONCLUSION

Second trimester tubal ectopic pregnancies are rare but carry a significant maternal mortality risk. Tubal ectopic pregnancies that progress beyond the first trimester are even more rare especially after receiving prenatal care. Despite being difficult to diagnose it should be considered and investigated regardless of prior ultrasound documentation in a pregnant female presenting with severe abdominal pain.
